# Inactivation of Salmonella on tainted foods: using blue light to disinfect cucumbers and processed meat products

**DOI:** 10.1002/fsn3.354

**Published:** 2016-03-10

**Authors:** J. Stephen Guffey, William C. Payne, Susan D. Motts, Pam Towery, Todd Hobson, Grafton Harrell, Logan Meurer, Kristoffer Lancaster

**Affiliations:** ^1^ Department of Physical TherapyArkansas State UniversityP.O. Box 910State UniversityArkansas; ^2^ Department of Clinical Laboratory ScienceArkansas State UniversityP.O. Box 910State UniversityArkansas; ^3^ Department of Nutritional SciencesArkansas State UniversityP.O. Box 910State UniversityArkansas

**Keywords:** Blue light, *E. coli*, food contamination, foodborne illness, *Salmonella*

## Abstract

Foodborne illness resulting from infectious organisms occurring in vegetables and processed meat is a serious health concern in the United States. Improved and cost‐effective techniques for disinfection are needed. Visible light in the blue range (405 nm) was administered to processed meat that had been inoculated with *Escherichia coli*. One application of light energy at doses of 10, 30, 60, and 100 J/cm^2^ was applied, in vitro. In the case of vegetables contaminated with *Salmonella* (cucumbers), 464 nm light was used at 6, 12, and 18 J/cm^2^. In both cases, after 20 hours of incubation, colony‐forming units were counted and compared to controls to determine whether the light energy inhibited growth of *E. coli* or *Salmonella*. *E. coli* – 405 nm light at doses of 30, 60, and 100 J/cm^2^ were all effective inhibitors of the organism. Kill rates of 75.61 – 96.34% were achieved. Salmonella – 464 nm light at doses of 6, 12, and 18 J/cm^2^ produced significant inactivation of the organism. Kill rates of 80.23–100% were obtained. Blue light, delivered in the wavelength/dose combinations used in this study is an effective inhibitor of both *E. coli* and *Salmonella* on actual foodstuffs. Blue light should be considered as a potentially effective tool in the effort to protect humans from foodborne illnesses.

## Introduction

Salmonella is a facultative anaerobic, gram‐negative bacterium (Capalonga et al. 2014). There are just two species of *Salmonella*,* Salmonella bongori* and *Salmonella enterica*. The latter is divided into six subspecies: *enterica*,* salamae*,* arizonae*,* diazonae*,* houtenase*, and *indica*. These subspecies are further divided into numerous serovars (Dieckmann et al. 2008). The species *Salmonella enterica* subsp. *enterica* contains over 60% of the total number of serovars and 99% of the serovars that are capable of infecting cold‐ and warm‐blooded animals, including humans. Infections are usually contracted from sources such as:

- Poultry, pork, beef, and fish (seafood), if the meat is prepared incorrectly or is infected with the bacteria after preparation (Mughini‐Gras et al. 2014).
- Infected eggs, egg products, and milk when not prepared, handled, or refrigerated properly (Mughini‐Gras et al. 2014).
- Tainted fruits and vegetables (Scallan et al. 2011).

Salmonellosis, the infection caused by the *Salmonella* bacteria results in more hospitalizations and deaths than any other foodborne illness (more than 1 million Americans contract salmonellosis yearly) (Jackson et al. 2013). Most people infected with *Salmonella* develop diarrhea, fever, vomiting, and abdominal cramps 12–72 h after infection. The illness typically lasts 4–7 days. Spontaneous recovery usually occurs without treatment (FoodSafety.gov, 2015). In some cases, the diarrhea may be so severe that the patient becomes dangerously dehydrated and must be hospitalized. The elderly, infants, and those with impaired immune systems are more likely to develop severe illness (Shimoni et al. 1999). Some people afflicted with salmonellosis later experience reactive arthritis, which can have long‐lasting, disabling effects (Ajene et al. 2013). *Salmonella* bacteria can survive for some time without a host; thus, they are frequently found in polluted water, with contamination from the excrement of carrier animals being particularly important (Waldner et al. 2012).

On November 19, 2015, the Centers for Disease Control and Prevention (CDC) reported a multistate outbreak of *Salmonella* Poona associated with imported cucumbers (Centers for Disease Control and Prevention, 2015a). Currently, 838 cases of *Salmonella* Poona have been reported in 38 states. One hundred and sixty‐five hospitalizations have been required for those infected and four deaths have occurred (Centers for Disease Control and Prevention, 2015a).

*Escherichia coli*, another bacterium in the family *Enterobacteriaceae* and a quite similar organism to *Salmonella*, is commonly referred to as the colon bacillus because of its prevalence in the gastrointestinal tract of humans and other animals. For decades, public health laboratories have used the presence of this and related organisms (coliforms) in sources of drinking water as a sign of fecal contamination and an indication that water is not fit for human consumption.

Most strains of *E. coli* are nonpathogens and a part of the normal microbiota of the gastrointestinal tract. They play a beneficial role by competing for space and nutrients, thereby preventing pathogenic bacteria from gaining a foothold in the colon and causing illness. Certain strains of *E. coli* have demonstrated the ability to cause a wide variety of diseases including urinary tract infections, respiratory tract infections, bacterial meningitis, neonatal sepsis, and diarrhea (Kiser et al. 2011).

As of November 23, 2015, 19 individuals from seven states have been reported to have fallen ill with a pathogenic strain of *E. coli* known to cause a gastrointestinal illness associated with loose, watery, and bloody stools. Serotype *E. coli* O57:H7 is one among other strains referred to as Shiga toxin‐producing *E. coli* (STEC). Transmission to humans has been associated with the ingestion of undercooked ground beef, unpasteurized apple cider, fresh fruits and vegetables, and contaminated water (Centers for Disease Control and Prevention, 2015b).

Current methods of food sanitization include thermal methods (high heat, refrigeration, freezing), ultraviolet irradiation, and application of chemical sanitizers (Butz and Tauscher 2002; Allende et al. 2008; Birmpa et al. 2013). While these methods can be effective, associated costs, loss of nutritional content, changes in taste and texture, and concerns that certain chemical sanitizers may be carcinogenic have made room for exploration into alternative means of achieving food safety.

In vitro inhibition of various bacteria using blue light (405–470 nm) has been frequently demonstrated (Guffey and Wilborn 2006a,b; Guffey et al. 2013a,b,c, 2014a,b,c,d,e,f). To our knowledge, only (Guffey et al. 2013a) have examined the potential for blue light to inhibit *Salmonella*. Using 470 nm blue light at high fluences (165 and 220 J/cm^2^), these researchers were able to demonstrate complete inactivation. The research by Guffey et al. (2013a) was probably the first demonstration that 470 nm blue light can inactivate *Salmonella*.

We propose to build upon the work of Bumah et al. (2015) by investigating whether blue light (464 nm) can disinfect cucumbers inoculated with *Salmonella*. Due to the similarities between *Salmonella* and *E. coli*, we further propose to determine whether blue light (405 nm) can be used to inactivate *E. coli* from processed meat. If it can be demonstrated that blue light can inactivate *Salmonella* and *E. coli* on actual foodstuffs, the potential benefit to the safety of the food chain, and therefore to human health, would be quite significant.

## Methods

### Preparation of organisms

The organisms used in this study included *Salmonella* serotype Typhimurium (or *S. typhimurium*) American Type Culture Collection (ATCC^®^) 14028 and *Escherichia coli* ATCC 25922 (ATCC, Manassas, VA). The organisms were grown on BBL tryptic soy agar II supplemented with 5% sheep blood (Becton, Dickinson and Company, Sparks, MD). After an overnight incubation at 35–37°, a sterile cotton‐tipped swab was used to remove 3–4 well isolated colonies. The organisms were then suspended in sterile deionized water and the turbidity adjusted to match a 0.5 McFarland standard, yielding an approximate cell density of 1.5 × 10^8^ CFU/mL. Using an adjustable automatic pipette (to maintain accuracy and reproducibility), the cell suspension was diluted 1/20,000. This final dilution rendered the culture density to be 7.5 × 10^3^ CFU/mL. All dilutions were made immediately before administration of the light.

### Preparation of food product

A ready‐to‐eat meat product (packaged hot dogs) was utilized as the growth substrate for *E. coli*. The meat was cut into small squares to fit into the bottom of a 60 × 15 mm sterile, polystyrene petri dish. A different hot dog was used for each plate. The surface of the cut meat product was blotted with a sterile, absorbent cotton square to ensure the surface was not excessively moist and to remove any oily residue. An adjustable automatic pipette, designed to deliver 0.5–10 microliters, was used to instill a 10 μL aliquot of the diluted bacterial suspension onto the surface of the meat product.

The growth substrate utilized for the *S. typhimurium* was the skin of a cucumber cut into small squares and placed into the bottom of a 60 × 15 mm sterile, polystyrene petri dish. Each plate was populated with skin from a separate cucumber. Incorporation of both meat and vegetable in our study reflects the current state of affairs wherein contaminated food stuffs include meats, vegetables, and fruit.

Early on, contamination from a variety of organisms including coliforms made an accurate colony count of *S. typhimurium* impossible. The contamination may have resulted from the fertilizers used to facilitate growth of such ground vegetables. It is also possible that contaminates were soil microbes that adhered to the outside of the cucumber. An initial cleansing of the skin of the cucumber with 70% alcohol resolved the contamination problem.

An adjustable automatic pipette, designed to deliver 0.5–10 *μ*L, was used to instill a 10 μL aliquot of the diluted bacterial suspension onto the surface of the sanitized cucumber skin.

### Light energy application

The substrates (both meat product and cucumber skin) were then illuminated to deliver a predetermined dose of 405 or 464 nm light energy. After delivery of the appropriate dose, the surface of the irradiated substrates were pressed against the surface of MacConkey agar (Becton, Dickinson and Company, Sparks, MD) in a 60 × 15 mm sterile, polystyrene petri dish. The substrates were removed and sterile, disposable, calibrated inoculating loops (10 μL size) were used to spread the inoculum in a “star streak” pattern. After incubation at 35–37°C for a period of approximately 20 h, the plates were examined for the presence of bacterial colonies and a colony count recorded.

We chose to illuminate the *E. coli* using supraluminous diode (SLD) light probes capable of delivering primary wavelengths of 405 nm. The probes consisted of a 5 cm^2^ illuminating surface area comprised of 34 SLDs with a maximum power output of 1000 milliwatts (rate of energy delivery of 83.3 milliwatts/cm^2^). Since output for the probes was held constant, adjustment in time of irradiation provided the doses used in the experiment (10, 30, 60, 90, and 120 J/cm^2^). The probes were fixed in a frame so as to be very near (3–5 mm) the surface of the inoculated meat product as the light energy was delivered.

Light energy was delivered to the *Salmonella* organism using light pads. A wavelength of 464 nm was delivered to the *Salmonella*. The 464 nm wavelength was chosen for this organism for two reasons. First, to closely (in terms of wavelength) approximate the work of Bumah et al. (2015). Secondly, we chose 464 nm because we wished to deliver the light energy in a more diffuse manner using light pads. We did not have light pads that would deliver 405 nm. The pads consisted of a 353 cm^2^ total illuminating surface area comprised of 176 SLDs with a maximum power output of 5160 milliwatts (rate of energy delivery of 16.6 milliwatts/cm^2^). Dose (fluence) was calculated in J/cm^2^. The inoculated sample was placed in a petri dish that was located between two light pads. Since output for the probe and the pads was held constant, adjustment in time of irradiation provided the dose.

## Results

### Salmonella

**Figure 1:**
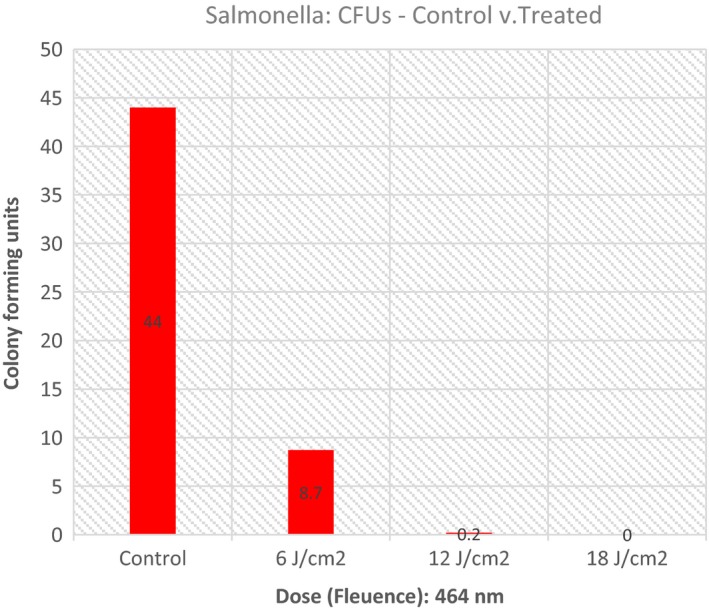
Colony‐forming units (CFUs) – control versus treated: *Salmonella*.

The null hypothesis for this experiment with regard to *Salmonella* was, “There will be no difference in the number of colony forming units in controls versus treated plates regardless of the dose applied using 464 nm light.” An a priori level for alpha was set at 0.05. Table 1 is provided to display specific descriptive data for each condition involved in the experiment. Figure 1 provides a graphical display of these data. The number of trials (*n*) for each condition was arbitrarily set by the investigators.

**Table 1:** Descriptives – number of colony‐forming units (CFUs) per condition ‐ *Salmonella*

CFUs								
	*N*	Mean	Std. deviation	Std. error	95% Confidence interval for mean		Minimum	Maximum
					Lower bound	Upper bound		
Control	11	40.5455	12.50891	3.77158	32.1419	48.9491	6.00	50.00
6 J/cm^2^	9	9.0000	3.64005	1.21335	6.2020	11.7980	2.00	13.00
12 J/cm^2^	10	0.2000	0.63246	0.20000	−0.2524	0.6524	0.00	2.00
18 J/cm^2^	10	0.0000	0.00000	0.00000	0.0000	0.0000	0.00	0.00
Total	40	13.2250	18.59899	2.94076	7.2768	19.1732	0.00	50.00

**Figure 2:**
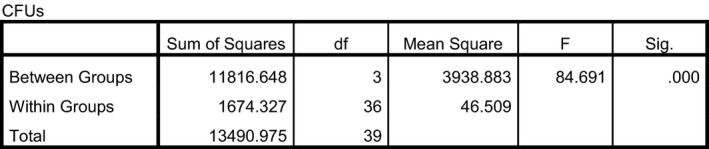
ANOVA – *Salmonella*.

A one‐way ANOVA was performed to determine whether there were any conditions (doses) that were different from the others in terms of colony‐forming units. Mean colony‐forming units were compared for each group (Control vs. Treated). The ANOVA indicated a significant difference was present (*P* ≤ 0.0001). Figure 2 demonstrates this analysis.

**Figure 3:**
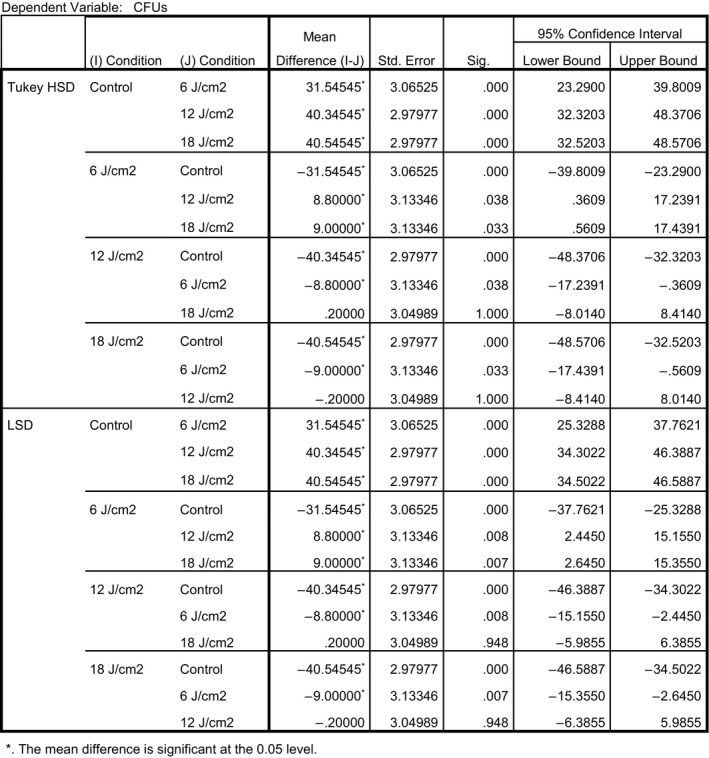
Multiple comparisons (least significant difference and Tukey's honest significant difference).

Two different post hoc analyses [least significant difference (LSD) and Tukey Honest Significant Difference (HSD)] were employed to identify which conditions produced an outcome that would allow a rejection of the null hypothesis. Each of the post hoc analyses demonstrated that all treatment conditions were significantly different from the control. Each of the treatment conditions effectively inactivated the *Salmonella*. Additionally, 12 and 18 J/cm^2^ were both significantly different from 6 J/cm^2^, but 12 and 18 were not different from each other. Figure 3 provides precise details related to the post hoc analyses.

### *Escherichia coli*

**Figure 4:**
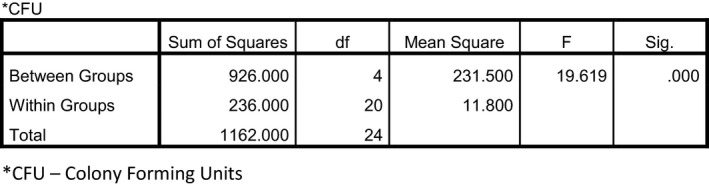
ANOVA – *E. Coli*.

Just as in the case for *Salmonella*, the null hypothesis for this experiment with regard to *E. coli* was, “There will be no difference in the number of colony forming units in controls versus treated plates regardless of the dose applied using 464 nm light.” An a priori level for alpha was set at 0.05. Table 2 is provided to display specific descriptive data for each condition involved in the experiment. The number of trials (*n*) for each condition was arbitrarily set by the investigators. Figure 4 summarizes these data in a graphical display.

**Table 2:** Descriptives – number of colony‐forming units (CFUs) per condition – *E. coli*

CFU								
	*N*	Mean	Std. deviation	Std. error	95% Confidence interval for mean		Minimum	Maximum
					Lower bound	Upper bound		
Control	5	16.4000	6.18870	2.76767	8.7157	24.0843	8.00	23.00
10 J/cm^2^	5	10.2000	3.42053	1.52971	5.9529	14.4471	6.00	15.00
30 J/cm^2^	5	4.0000	2.73861	1.22474	0.5996	7.4004	0.00	7.00
60 J/cm^2^	5	0.8000	0.83666	0.37417	−0.2389	1.8389	0.00	2.00
100 J/cm^2^	5	0.6000	0.89443	0.40000	−0.5106	1.7106	0.00	2.00
Total	25	6.4000	6.95821	1.39164	3.5278	9.2722	0.00	23.00

**Figure 5:**
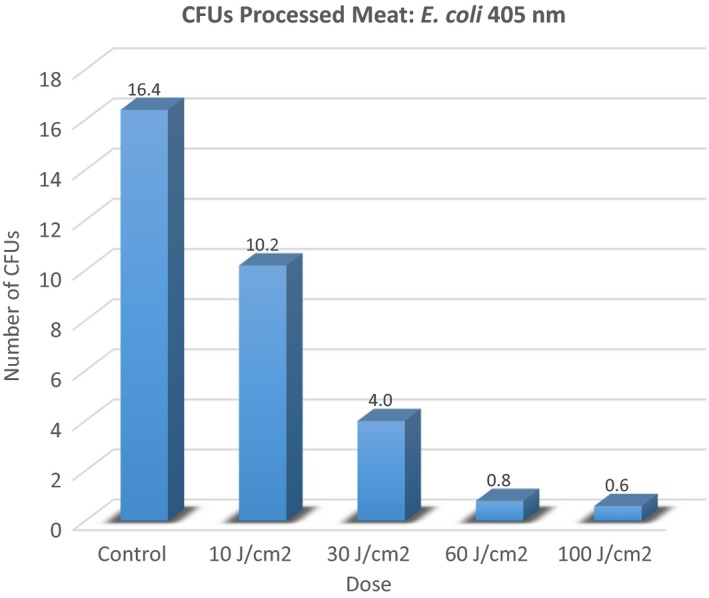
Colony‐forming units (CFUs) – control versus treated: *E. Coli*.

Again, as with data from the *Salmonella* experiment, a one‐way ANOVA was performed to determine whether there were any conditions (doses) that were different from the others in terms of colony‐forming units. Mean colony‐forming units were compared for each group (Control vs. Treated). The ANOVA indicated a significant difference was present (*P* ≤ 0.0001). Figure 5 demonstrates this analysis.

**Figure 6:**
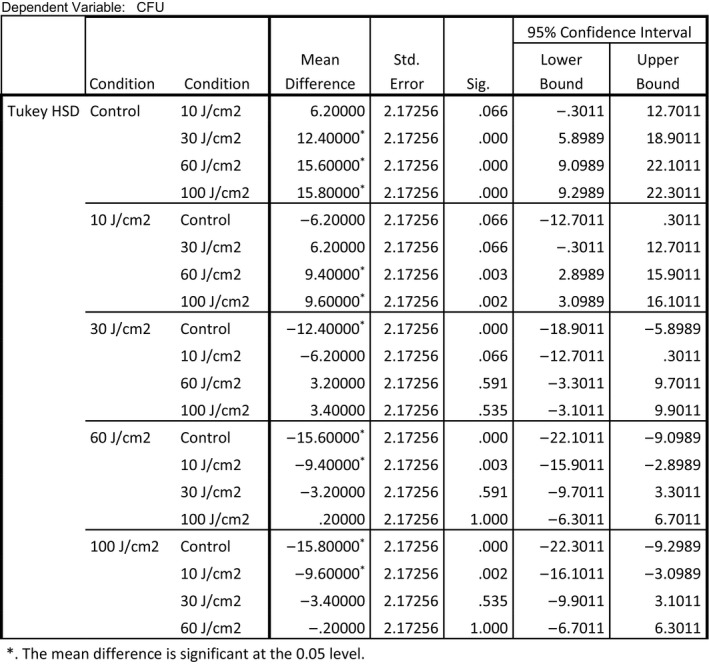
Multiple comparisons (Tukey's honest significant difference ‐ HSD).

**Figure 7:**
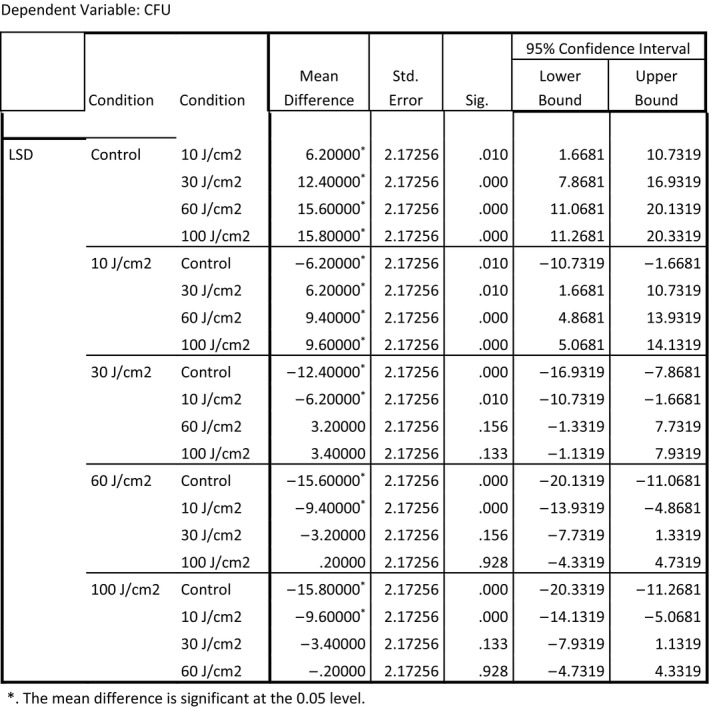
Multiple comparisons (least significant difference ‐ LSD).

Two different post hoc analyses LSD and Tukey Honest Significant Difference [HSD]) were employed to identify which conditions produced an outcome that would allow a rejection of the null hypothesis. Each of the post hoc analyses demonstrated that all treatment conditions were significantly different from the control except for 10 J/cm^2^. The Tukey HSD indicated that 10 J/cm^2^ did not provide significant inactivation. The LSD post hoc test indicated that all treatment conditions were effective for inactivation of *E. coli*. All the other treatment conditions (30, 60, and 100 J/cm^2^ were demonstrated effective for inactivation of *E. coli*. Figures 6 and 7 display data related to these post hoc tests.

## Discussion

**Figure 8:**
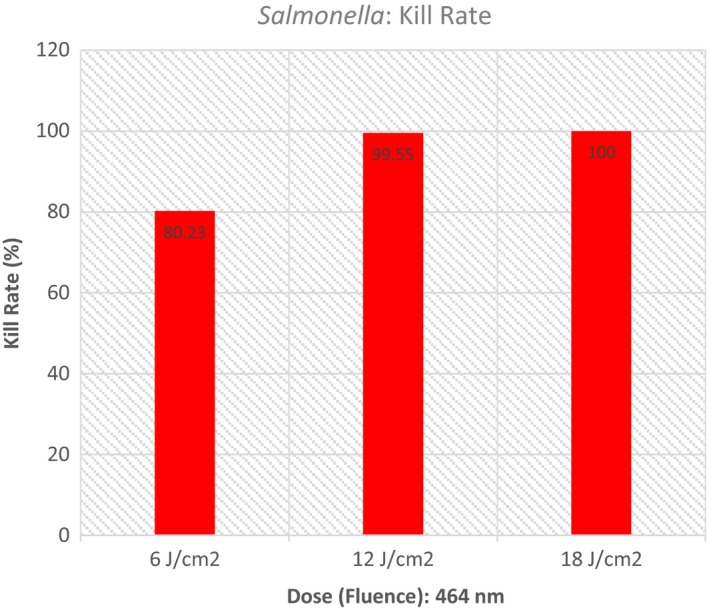
Inactivation (kill) rates for *Salmonella*.

**Figure 9:**
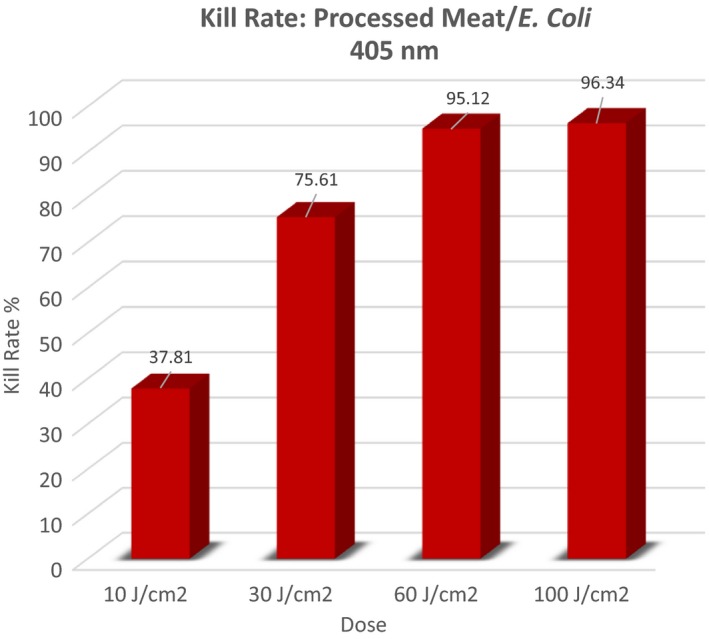
Inactivation (kill) rates for *E. coli*.

Our results demonstrate that *E. coli* and *Salmonella* can be inactivated by blue light. Blue light at 405 nm was the best energy source for *E. coli* inactivation, while *Salmonella* was most affected by 464 nm energy (See Figures 8 and 9). Generally, our results are in line with those of Bumah et al. (2015) with regard to Salmonella. In the case of our experiment, inactivation was achieved with considerably lower fluences. Bumah et al. (2015) reported fluences of over 100 J/cm^2^ to be required to completely inactivate. We were able to achieve complete inactivation of Salmonella, using 464 nm light at 18 J/cm^2^ and very nearly complete inactivation of *E. coli* at 60 J/cm^2^. See Figures 8 and 9 for a graphical depiction of these points.

It is important to consider three points when comparing the results from this study to those reported by Bumah et al. (2015). First, we irradiated actual foodstuffs that had been inoculated with the respective organisms. Salmonella was inoculated to cucumber peels, and *E. coli* was inoculated on processed meat (hot dogs). The second point to consider is the density of the cultures used. Bumah et al. (2015) reported a culture density of 5 × 10^6^ CFU/mL. The density used in our experiment was 7.5 × 10^3^ CFU/mL. Bumah et al. (2013) have previously demonstrated that density of culture can influence relative inactivation rates. Differences in culture density could account for the effect we obtained at lower fluences. The third point relates to rate of delivery (irradiance). Bumah et al. (2015) delivered blue light to *Salmonella* using a higher irradiance (30 mW/cm^2^) than was employed in our research. We delivered the 464 nm light to Salmonella inoculated on cucumber peels at 16.6 mW/cm^2^.

It is important to point out that the results we obtained were very much akin to those of (Bumah et al. 2015), we simply saw these results occur at lower fluences. It is generally accepted that the mechanism of cell death brought on by blue light is the photoexcitation of endogenous porphyrins, leading to the production of reactive oxygen species (ROS) that cause the death of the bacteria when the wavelength used is 405 nm (Hamblin and Demidova 2006). Because both *E. coli* and *Salmonella* are gram‐negative species and generally have fewer endogenous porphyrins than gram‐positive bacteria, Bumah et al. (2015) proposed higher fluences would be necessary to completely eliminate gram‐negative bacteria. We agree that this is a valid point. In the case of *E. coli,* relatively high fluences were required when the 405 nm wavelength was employed. For Salmonella, we elected to make two adjustments. We lowered the irradiance and used a 464 nm wavelength. A second understood mechanism of cell death is facilitation of flavins. It is possible that the bactericidal action was a function of the stimulation of flavins since peak activation of endogenous flavins is known to occur when 470 nm light is used (De Lucca et al. 2012). It is also possible that the level of irradiance played a role. This point needs further investigation.

Whether our results were affected by the condition of actual foodstuffs, the density of the culture, or a function of the irradiance, blue light holds great potential to inactivate microorganisms frequently contaminating the food supply. We are confident in coming to the following conclusions.

Blue light (405 nm) is an effective inhibitor of *E. coli* located on ready‐to‐eat processed meat. Doses (fluences) from 30 to 100 J/cm^2^ can produce from 75% to near 100% inactivation of this organism in these conditions.

Blue light (464 nm) is an effective inhibitor of Salmonella on vegetables (cucumbers). Using fluences as low as 6 J/cm^2^, an 80% inactivation can be achieved. A fluence of 18 J/cm^2^ can completely eliminate *Salmonella* from cucumbers.

This research confirms the work of Bumah et al. (2015). Moreover, it demonstrates that blue light can disinfect actual foodstuffs. There is evidence that blue light should be considered as a tool in the prevention of foodborne illnesses.

## Conflict of Interest

None declared.
